# Dexmedetomidine Use and Short-Term All-Cause Mortality in Intensive Care Unit Patients with Ischemic Stroke and Atrial Fibrillation: A Propensity-Matched Analysis from MIMIC-IV

**DOI:** 10.1159/ene/acsag008

**Published:** 2026-10-15

**Authors:** Zengyan Bai, Ke Shi, Hongwei Liu, Zhinan Ye, Suqian Xu

**Affiliations:** a Department of Critical Care Medicine, Taiyuan City Central Hospital, The Ninth Clinical Medical College of Shanxi Medical University, Taiyuan, China; b Department of Neurology, Xiangshui County Peopleʼs Hospital, Yancheng, China; c Department of Neurology, Taiyuan City Central Hospital, The Ninth Clinical Medical College of Shanxi Medical University, Taiyuan, China; d Department of Neurology, Taizhou Municipal Hospital (Taizhou University Affiliated Municipal Hospital), School of Medicine, Taizhou University, Taizhou, China

**Keywords:** Dexmedetomidine, Ischemic stroke, Atrial fibrillation, Intensive care unit, Propensity score matching

## Abstract

**Introduction:**

Preclinical studies have demonstrated that dexmedetomidine (DEX), a sedative widely used in intensive care units (ICU), exerts neuroprotective effects. However, its potential protective role and underlying mechanisms in ischemic stroke patients with atrial fibrillation (IS-AF) remain unclear. The studyʼs objective was to assess the connection between the early administration of DEX and short-term mortality from all causes in patients with IS-AF.

**Methods:**

From the MIMIC-IV database, patients with IS-AF were identified, including those who were administered DEX and those who were not. The primary endpoint was 7-day all-cause mortality, and the secondary endpoint was 90-day all-cause mortality. Propensity score matching (PSM) was utilized to ensure baseline covariates were balanced. Cox proportional hazards models and Kaplan-Meier survival curves and were utilized to evaluate the association between DEX use and mortality, with subgroup analyses conducted to ensure the findingsʼ robustness.

**Results:**

In total, 1,355 patients with IS-AF participated, with 253 (18.7%) receiving DEX. After PSM, 208 pairs were matched. In the fully adjusted model, DEX administration was significantly associated with lower all-cause mortality compared with the control group at both 7 days (before PSM, HR = 0.36, 95% CI: 0.19–0.68, *p* = 0.002; after PSM, HR = 0.26, 95% CI: 0.12–0.54, *p* < 0.001) and 90 days (before PSM, HR = 0.84, 95% CI: 0.66–1.07, *p* = 0.162; after PSM, HR = 0.72, 95% CI: 0.53–0.97, *p* = 0.031). The subgroup analyses revealed that DEXʼs protective effect was stronger in patients aged 70 and above, females, those with hypertension, and those without heart failure or chronic kidney disease. Kaplan-Meier survival curves consistently illustrated that DEX users had greater survival probabilities both before and after matching.

**Conclusion:**

In patients with IS-AF, DEX administration is connected to decreased all-cause mortality mortality, underlining the possible importance of DEX in ICU settings.

## Introduction

Worldwide, stroke is the second leading cause of death and the third leading cause of long-term disability, defined by a sudden onset of focal neurological deficits lasting over 24 h [1, 2]. Based on etiology, stroke can be classified into ischemic stroke and hemorrhagic stroke, accounting for approximately 87% and 13% of all strokes, respectively [3, 4]. In 2016, an estimated 5.5 million people died from stroke globally, and stroke-related disability-adjusted life years exceeded 116 million, with approximately 70% of stroke events and 87% of stroke-related deaths occurring in low- and middle-income countries [5].

In ischemic stroke, arterial occlusion is the central pathogenic mechanism, typically resulting from local thrombosis or atherosclerosis, but may also be caused by distal emboli blocking cerebral arteries or lacunar infarctions secondary to small vessel disease. Individuals with conditions like atrial fibrillation (AF), myocardial infarction, prosthetic heart valves, rheumatic heart disease, or severe carotid stenosis are at a higher risk of experiencing embolic strokes [6]. The incidence of AF, the most widespread sustained cardiac arrhythmia, grows as people get older [7, 8]. It is a well-recognized risk factor for thromboembolism and the primary cause of cardioembolic stroke. AF promotes blood stasis and thrombus formation, substantially elevating the risk of cerebrovascular events, including transient ischemic attacks and ischemic stroke [9, 10]. Despite the widespread use of anticoagulation therapy and control of systolic blood pressure, which reduced AF-related stroke incidence between 1980 and 2000 [11], ischemic stroke with AF (IS-AF) still imposes a considerable clinical burden, characterized by more severe neurological deficits, higher mortality, and recurrence. Current risk assessment tools, such as the CHA_2_DS_2_-VASc score [12], have limited predictive accuracy, potentially leading to overtreatment in low-risk individuals.

In light of these developments, the pursuit of new interventions with dual neuroprotective and cardioregulatory functions has emerged as a crucial research focus in the IS-AF area. Dexmedetomidine (DEX), a highly selective α2-adrenergic receptor agonist, is commonly utilized for sedation in patients who are critically ill or undergoing surgery. According to the Society of Critical Care Medicineʼs PADIS guidelines, DEX is recommended as a non-benzodiazepine sedative for mechanically ventilated adults, particularly when light sedation and cooperation are desired or when delirium prevention is a clinical concern [13–15]. In neurocritical care, these characteristics may facilitate repeated neurological assessments while preserving spontaneous respiration. Beyond its sedative effects, DEX reduces central sympathetic activity and has demonstrated anti-inflammatory, neuroprotective, and cardioprotective properties [16–18]. These influences could be especially pertinent to critically ill patients with IS-AF, who usually exhibit sympathetic overactivation, tachyarrhythmias, and hemodynamic instability. Evidence suggests that DEX also provides heart protection as it reduced the size of myocardial infarctions and improved the contractile function of the heart in animal models [18]. These properties may be particularly relevant to critically ill patients with IS-AF, who often exhibit sympathetic overactivation, tachyarrhythmias, and hemodynamic instability, thereby providing a biological rationale for investigating the association between DEX use and clinical outcomes in this population.

However, the clinical impact of DEX in the IS-AF population has not been systematically investigated. Whether its dual neuro-cardiac effects can translate into tangible benefits, such as reduced stroke recurrence, improved neurological recovery, or decreased AF burden, remains to be elucidated. There is an urgent need to characterize the role of DEX in improving outcomes among patients with ischemic stroke complicated by AF, which may inform treatment strategies in this high-risk population. Propensity score matching (PSM) has been employed to mitigate sample selection bias and emulate randomized conditions [19, 20]. We leveraged the MIMIC-IV database in this study to assess the association between DEX application and clinical outcomes, with an emphasis on short-term all-cause mortality in patients suffering from IS-AF.

## Methods

### Data Foundation

A retrospective cohort analysis was performed in this study, employing the MIMIC-IV database (version 3.0), which holds patient records from intensive care unit (ICU), between 2008 and 2019. The collaboration between MIT and Beth Israel Deaconess Medical Center led to the development of MIMIC-IV, which has been approved by the by the Institutional Review Board (IRB). No further informed consent was required because all patient information is de-identified. Certification from the Collaborative Institutional Training Initiative (CITI, certification number 68968318) allowed our research team members to access and use the database. In compliance with ethical standards and patient privacy, all data extraction and analyses were carried out without affecting clinical care. Eligibility for inclusion was given to patients with AF who were admitted to the ICU for the first time following an ischemic stroke.

### Study Population

From the MIMIC-IV database, a total of 94,458 ICU admissions were initially examined. The study included 1,535 patients after excluding 9,216 with prior ICU stays and 83,738 without cerebrovascular disease or AF. The primary study cohort was finalized at 1,355 patients after excluding those aged under 18 (*n* = 12) or with ICU stays under 24 h (*n* = 137). Patients were stratified based on DEX exposure (exposed group: *n* = 253; unexposed group: *n* = 1,102). In order to minimize confounding factors, 1:1 PSM was executed, resulting in 208 matched pairs (*n* = 416) for later analyses. The study flowchart is presented in Figure 1.

**Fig. 1.**
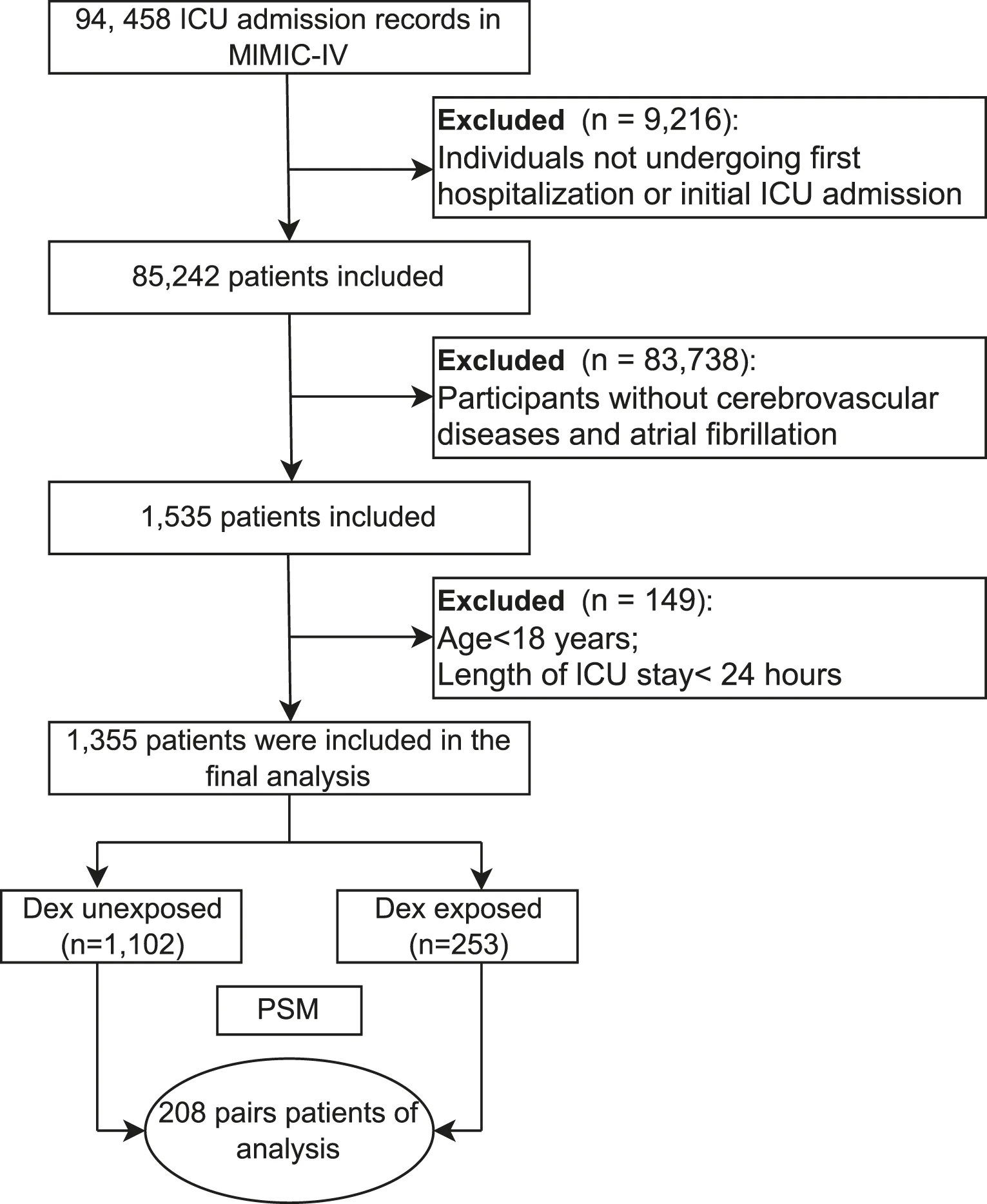
Flowchart of sample selection. Dex, dexmedetomidine; PSM, propensity score matching; ICU, intensive care unit.

### Data Extraction

The extraction of data on DEX administration and mortality outcomes during ICU hospitalization was performed using SQL. Clinical data were meticulously collected to examine the relationship between DEX administration and death rates in patients with acute ischemic stroke complicated by AF. Variables included demographic characteristics (age, sex, and body weight); vital signs (heart rate, systolic and diastolic blood pressure, mean arterial pressure, respiratory rate, body temperature, and peripheral oxygen saturation); and laboratory parameters (international normalized ratio and activated partial thromboplastin time). Comorbidities, including heart failure, chronic kidney disease, diabetes, hypertension, chronic pulmonary disease, malignancy, and moderate-to-severe liver disease, were documented. Information on therapeutic interventions, including continuous renal replacement therapy (CRRT), mechanical ventilation, vasopressor use, antiplatelet and anticoagulation therapy, and thrombolysis, was also recorded.

### Ascertainment of DEX Administration

In routine intensive care practice, DEX is administered as a continuous intravenous infusion, typically initiated at maintenance doses ranging from 0.2 to 0.7 μg/kg/h and titrated according to predefined sedation targets and hemodynamic tolerance. For some cases, an initial dose ranging from 0.5 to 1.0 μg/kg over a period of 10–20 min may be applied, depending on the individualʼs clinical situation. Infusion rates are regulated by sedation scales and cardiovascular parameters, and the duration of administration is dependent on the patientʼs response and clinical indication.

For the purposes of this study, DEX exposure was identified as any instance of DEX being administered during an ICU stay. Exposure status was determined based on recorded medication administration data. Given the heterogeneity in dosing regimens, infusion duration, and timing of initiation in routine practice, DEX use was analyzed as a dichotomous variable (DEX vs. no DEX). No stratification was performed according to cumulative dose, infusion duration or timing relative to stroke onset.

### Clinical Outcomes

The chief endpoint was mortality from any cause within 7 days after entering the ICU, with a secondary endpoint being mortality from any cause within 90 days of ICU entry.

### Statistical Analysis

The missForest packageʼs random forest algorithm was employed to impute variables with fewer than 20% missing data (online suppl. Table 1; for all online suppl. material, see https://doi.org/10.1159/ene/acsag008). Medians with interquartile ranges (Q1-Q3) summarized continuous variables, while categorical variables were expressed in terms of proportions. To evaluate baseline differences between the DEX and non-DEX groups, continuous variables were tested with the independent-samples *t* test or Mann-Whitney U test, and categorical data were analyzed using Pearson’s chi-square test. Using Cox proportional hazards regression, the study assessed the link between DEX administration and clinical outcomes, producing hazard ratios (HR) and 95% confidence intervals (CI) after considering relevant confounders. Prior to multivariable Cox regression, multicollinearity was assessed using variance inflation factors, and all variables had variance inflation factors below 5, with the maximum being 4.3 for SAPSII (online suppl. Table 2). To balance baseline differences, 1:1 PSM was utilized with nearest-neighbor matching and a caliper of 0.1 standard deviations of the logit of the propensity score. In observational studies, where randomization is impractical, PSM helps mitigate confounding by ensuring baseline covariates are balanced between treatment and control groups. To ensure better comparability, individuals were matched by similar propensity scores and those not matched were left out. Standardized mean differences (SMDs) were used to evaluate PSMʼs effectiveness, with an SMD of 0.1 or less indicating a good balance of baseline characteristics. Before and after PSM, Kaplan-Meier survival analysis and Cox regression were employed to compare outcomes between DEX users and non-users. In the matched cohort, the relationship between DEX use and all-cause mortality at 7 and 90 days was assessed using univariable and stepwise multivariable Cox regression models. For the multivariable models, variables with *p* values lower than 0.05 in the univariable analysis were included (online suppl. Table 3). Three hierarchical models were constructed: Model 1 was unadjusted; Model 2 was adjusted for age, CCI, SAPSII, and OASIS; and Model 3 was further adjusted for treatment-related interventions, including anti platelet agents, anticoagulant drugs and thrombolysis. *R* software (version 4.3.3, 2024-02–29) and Zstats 1.0 (www.zstats.net) were used for all statistical analyses, and two-sided *p* values below 0.05 were treated as statistically significant.

## Results

### Baseline Characteristics

The study cohort consisted of 1,355 eligible patients with ischemic stroke and atrial fibrillation (IS-AF), of which 18.67% (*n* = 253) were given DEX during their ICU admission (Table 1). In comparison to non-DEX patients, individuals in the DEX group were younger, more often female, and had increased body weight, heart rate, body temperature, and oxygen saturation, but their systolic, diastolic, and mean arterial pressures were reduced. The DEX group exhibited elevated scores in SOFA, GCS, CCI, APSIII, SAPSII, and OASIS, and higher international normalized ratio and partial thromboplastin time values, while having a lower occurrence of diabetes, hypertension, and chronic pulmonary disease. In terms of therapeutic interventions, the DEX group experienced reduced rates of mechanical ventilation, vasopressor use, antiplatelet therapy, anticoagulation, and thrombolysis but had an increased use of CRRT. After applying PSM, 416 patients were selected for further analysis, with 208 in each group, and most baseline imbalances were addressed (Table 2). Overall, baseline characteristics, including age, sex, body weight, vital signs, and severity scores (SOFA, SAPSII, OASIS), were effectively balanced by PSM, with all *p* values >0.05 and SMDs nearly zero (Table 2).

**Table 1.** Characteristics of patients at baseline before PSM

Characteristics	Total (*n* = 1,355)	Non-Dex (*n* = 1,102)	Dex (*n* = 253)	*p* value	SMD
Age (years)	78.38 (69.56, 85.52)	79.81 (71.47, 86.87)	71.49 (64.54, 79.91)	<0.001	−0.592
Gender, *n* (%)	​	​	​	<0.001	​
Female	722 (53.28)	624 (56.62)	98 (38.74)	​	−0.367
Male	633 (46.72)	478 (43.38)	155 (61.26)	​	0.367
Weight (kg)	75.20 (63.00, 90.00)	74.00 (62.00, 88.80)	83.10 (70.00, 98.30)	<0.001	0.324
HR (beats/min)	104.00 (89.00, 122.00)	103.00 (88.00, 121.00)	107.00 (90.00, 127.00)	0.010	0.202
SBP (mm Hg)	159.00 (144.00, 175.00)	161.00 (147.00, 176.00)	149.00 (135.00, 169.00)	<0.001	−0.259
DBP (mm Hg)	98.00 (82.00, 114.00)	99.00 (85.00, 115.00)	90.00 (77.00, 105.00)	<0.001	−0.301
MBP (mm Hg)	112.00 (100.00, 128.00)	114.00 (102.00, 128.00)	105.00 (95.00, 123.00)	<0.001	−0.218
RR (breaths/min)	28.00 (24.00, 31.00)	27.00 (24.00, 31.00)	28.00 (24.00, 33.00)	0.188	0.125
T (°C)	37.22 (37.00, 37.67)	37.22 (37.00, 37.60)	37.39 (37.06, 37.94)	<0.001	0.245
SPO2 (%)	100.00 (99.00, 100.00)	100.00 (99.00, 100.00)	100.00 (100.00, 100.00)	<0.001	0.396
SOFA	4.00 (2.00, 6.00)	3.00 (2.00, 5.00)	6.00 (4.00, 9.00)	<0.001	0.748
GCS	14.00 (11.00, 15.00)	14.00 (11.00, 15.00)	15.00 (12.00, 15.00)	<0.001	0.111
CCI	8.00 (6.00, 9.00)	8.00 (6.00, 9.00)	7.00 (6.00, 9.00)	0.076	−0.135
APSIII	42.00 (31.00, 54.00)	40.00 (30.00, 52.00)	50.00 (39.00, 67.00)	<0.001	0.548
SAPSII	37.00 (31.00, 46.00)	36.00 (30.00, 44.00)	43.00 (35.00, 55.00)	<0.001	0.543
OASIS	34.00 (28.00, 40.00)	33.00 (28.00, 39.00)	37.00 (32.00, 43.00)	<0.001	0.481
INR	1.30 (1.20, 1.56)	1.30 (1.20, 1.50)	1.40 (1.20, 1.70)	<0.001	0.157
PTT (s)	31.60 (27.60, 43.79)	31.31 (27.40, 42.80)	33.30 (28.70, 47.10)	0.009	0.119
CKD, *n* (%)	​	​	​	0.260	​
No	1,033 (76.24)	847 (76.86)	186 (73.52)	​	−0.076
Yes	322 (23.76)	255 (23.14)	67 (26.48)	​	0.076
Diabetes, *n* (%)	​	​	​	0.001	​
No	898 (66.27)	752 (68.24)	146 (57.71)	​	−0.213
Yes	457 (33.73)	350 (31.76)	107 (42.29)	​	0.213
Hypertension, *n* (%)	​	​	​	0.038	​
No	713 (52.62)	565 (51.27)	148 (58.50)	​	0.147
Yes	642 (47.38)	537 (48.73)	105 (41.50)	​	−0.147
Heart failure, *n* (%)	​	​	​	0.072	​
No	828 (61.11)	686 (62.25)	142 (56.13)	​	−0.123
Yes	527 (38.89)	416 (37.75)	111 (43.87)	​	0.123
Malignancy, *n* (%)	​	​	​	0.194	​
No	1,248 (92.1)	1,020 (92.56)	228 (90.12)	​	−0.082
Yes	107 (7.9)	82 (7.44)	25 (9.88)	​	0.082
Chronic pulmonary disease, *n* (%)	​	​	​	0.045	​
No	1,109 (81.85)	913 (82.85)	196 (77.47)	​	−0.129
Yes	246 (18.15)	189 (17.15)	57 (22.53)	​	0.129
Moderate to severe liver disease, *n* (%)	​	​	​	0.541	​
No	1,341 (98.97)	1,092 (99.09)	249 (98.42)	​	−0.054
Yes	14 (1.03)	10 (0.91)	4 (1.58)	​	0.054
Thrombolysis, *n* (%)	​	​	​	<0.001	​
No	1,162 (85.76)	989 (89.75)	173 (68.38)	​	−0.459
Yes	193 (14.24)	113 (10.25)	80 (31.62)	​	0.459
Anti-platelet agents, *n* (%)	​	​	​	0.479	​
No	356 (26.27)	294 (26.68)	62 (24.51)	​	−0.051
Yes	999 (73.73)	808 (73.32)	191 (75.49)	​	0.051
Anticoagulant drugs, *n* (%)	​	​	​	0.040	​
No	80 (5.9)	72 (6.53)	8 (3.16)	​	−0.193
Yes	1,275 (94.1)	1,030 (93.47)	245 (96.84)	​	0.193
CRRT, *n* (%)	​	​	​	<0.001	​
No	1,289 (95.13)	1,078 (97.82)	211 (83.40)	​	−0.388
Yes	66 (4.87)	24 (2.18)	42 (16.60)	​	0.388
Vasoactive agent, *n* (%)	​	​	​	<0.001	​
No	1,057 (78.01)	912 (82.76)	145 (57.31)	​	−0.514
Yes	298 (21.99)	190 (17.24)	108 (42.69)	​	0.514
Mechanical ventilation, *n* (%)	​	​	​	<0.001	​
No	789 (58.23)	749 (67.97)	40 (15.81)	​	−1.430
Yes	566 (41.77)	353 (32.03)	213 (84.19)	​	1.430

SOFA, sequential organ failure assessment; GCS, glasgow coma scale; CCI, charlson comorbidity index; SAPSII, simplified acute physiology score II; OASIS, oxford acute severity of illness score; INR, international normalized ratio; PTT, partial thromboplastin time; CKD, chronic kidney disease; HR, heart rate; SBP, systolic blood pressure; DBP, diastolic blood pressure; MBP, mean blood pressure; RR, respiratory rate; T, temperature; SPO2, oxygen saturation; CRRT, continuous renal replacement therapy; PSM, propensity score matching.

**Table 2.** Characteristics of patients at baseline after PSM

Characteristics	Total (*n* = 416)	Non-Dex (*n* = 208)	Dex (*n* = 208)	*p* value	SMD
Age (years)	72.78 (65.59, 81.67)	72.99 (65.59, 81.84)	71.75 (65.67, 81.24)	0.440	−0.051
Gender, *n* (%)	​	​	​	0.842	​
Female	172 (41.35)	85 (40.87)	87 (41.83)	​	0.019
Male	244 (58.65)	123 (59.13)	121 (58.17)	​	−0.019
Weight (kg)	80.32 (68.97, 93.62)	80.42 (68.44, 92.06)	80.15 (69.07, 95.89)	0.919	−0.005
HR (beats/min)	106.00 (89.00, 126.25)	106.00 (90.00, 127.00)	106.00 (89.00, 124.00)	0.670	−0.047
SBP (mm Hg)	153.00 (138.00, 169.00)	154.00 (139.00, 167.25)	150.00 (134.75, 170.25)	0.594	−0.031
DBP (mm Hg)	91.00 (77.00, 107.00)	92.00 (77.00, 107.25)	90.50 (77.00, 106.25)	0.625	−0.028
MBP (mm Hg)	106.00 (95.75, 127.25)	108.00 (95.75, 126.12)	105.50 (95.75, 130.25)	0.889	−0.018
RR (breaths/min)	28.00 (24.00, 32.00)	28.00 (25.00, 32.00)	27.50 (24.00, 33.00)	0.545	−0.069
T (°C)	37.39 (37.05, 37.90)	37.39 (37.00, 37.89)	37.43 (37.06, 37.91)	0.489	0.057
SPO2 (%)	100.00 (100.00, 100.00)	100.00 (100.00, 100.00)	100.00 (100.00, 100.00)	0.977	−0.013
SOFA	6.00 (3.00, 8.00)	6.00 (3.00, 9.00)	6.00 (3.00, 8.00)	0.841	−0.089
GCS	15.00 (11.75, 15.00)	14.50 (12.00, 15.00)	15.00 (11.00, 15.00)	0.610	0.010
CCI	7.00 (6.00, 9.00)	8.00 (6.00, 9.00)	7.00 (6.00, 9.00)	0.564	−0.059
APSIII	48.00 (36.00, 64.00)	49.00 (34.00, 65.00)	48.00 (38.00, 63.25)	0.878	−0.061
SAPSII	42.00 (33.75, 52.00)	42.00 (33.00, 52.00)	42.00 (34.00, 51.00)	0.936	−0.020
OASIS	37.00 (32.00, 43.00)	38.00 (32.00, 44.00)	37.00 (32.00, 42.00)	0.456	−0.062
INR	1.40 (1.20, 1.70)	1.30 (1.20, 1.60)	1.40 (1.20, 1.70)	0.692	−0.036
PTT (s)	32.95 (28.30, 45.30)	33.00 (27.78, 45.30)	32.35 (28.58, 44.57)	0.838	−0.016
CKD, *n* (%)	​	​	​	0.098	​
No	303 (72.84)	144 (69.23)	159 (76.44)	​	0.170
Yes	113 (27.16)	64 (30.77)	49 (23.56)	​	−0.170
Diabetes, *n* (%)	​	​	​	0.691	​
No	242 (58.17)	119 (57.21)	123 (59.13)	​	0.039
Yes	174 (41.83)	89 (42.79)	85 (40.87)	​	−0.039
Hypertension, *n* (%)	​	​	​	0.621	​
No	233 (56.01)	119 (57.21)	114 (54.81)	​	−0.048
Yes	183 (43.99)	89 (42.79)	94 (45.19)	​	0.048
Heart failure, *n* (%)	​	​	​	0.693	​
No	232 (55.77)	114 (54.81)	118 (56.73)	​	0.039
Yes	184 (44.23)	94 (45.19)	90 (43.27)	​	−0.039
Malignancy, *n* (%)	​	​	​	0.605	​
No	379 (91.11)	191 (91.83)	188 (90.38)	​	−0.049
Yes	37 (8.89)	17 (8.17)	20 (9.62)	​	0.049
Chronic pulmonary disease, *n* (%)	​	​	​	0.417	​
No	319 (76.68)	156 (75.00)	163 (78.37)	​	0.082
Yes	97 (23.32)	52 (25.00)	45 (21.63)	​	−0.082
Moderate to severe liver disease, *n* (%)	​	​	​	1.000	​
No	410 (98.56)	205 (98.56)	205 (98.56)	​	0.000
Yes	6 (1.44)	3 (1.44)	3 (1.44)	​	0.000
Thrombolysis, *n* (%)	​	​	​	0.910	​
No	313 (75.24)	157 (75.48)	156 (75.00)	​	−0.011
Yes	103 (24.76)	51 (24.52)	52 (25.00)	​	0.011
Anti-platelet agents, *n* (%)	​	​	​	0.550	​
No	89 (21.39)	42 (20.19)	47 (22.60)	​	0.057
Yes	327 (78.61)	166 (79.81)	161 (77.40)	​	−0.057
Anticoagulant drugs, *n* (%)	​	​	​	0.610	​
No	16 (3.85)	9 (4.33)	7 (3.37)	​	−0.053
Yes	400 (96.15)	199 (95.67)	201 (96.63)	​	0.053
CRRT, *n* (%)	​	​	​	1.000	​
No	378 (90.87)	189 (90.87)	189 (90.87)	​	0.000
Yes	38 (9.13)	19 (9.13)	19 (9.13)	​	0.000
Vasoactive agent, *n* (%)	​	​	​	0.921	​
No	243 (58.41)	121 (58.17)	122 (58.65)	​	0.010
Yes	173 (41.59)	87 (41.83)	86 (41.35)	​	−0.010
Mechanical ventilation, *n* (%)	​	​	​	0.901	​
No	81 (19.47)	41 (19.71)	40 (19.23)	​	−0.012
Yes	335 (80.53)	167 (80.29)	168 (80.77)	​	0.012

SOFA, sequential organ failure assessment; GCS, glasgow coma scale; CCI, charlson comorbidity index; SAPSII, simplified acute physiology score II; OASIS, oxford acute severity of illness score; INR, international normalized ratio; PTT, partial thromboplastin time; CKD, chronic kidney disease; HR, heart rate; SBP, systolic blood pressure; DBP, diastolic blood pressure; MBP, mean blood pressure; RR, respiratory rate; T, temperature; SPO2, oxygen saturation; CRRT, continuous renal replacement therapy; PSM, propensity score matching.

In online supplementary Figure 1, kernel density plots display the overlap of propensity score distributions for the DEX and non-DEX groups prior to and following matching. The non-DEX group had a unimodal distribution that was different from the overall mean before matching, while the DEX group had a multimodal distribution, pointing to significant initial differences. Post-matching, the distributions were mostly aligned, primarily concentrated between 0.25 and 0.5, with a peak around 0.4, indicating strong matching quality and comparability. Online supplementary Figure 2 presents the SMDs for baseline covariates pre- and post-matching, where an SMD less than 0.1 signifies adequate balance. In online supplementary Figure 1, the overall distribution balance is considered, whereas online supplementary Figure 2 highlights the balance for certain variables. Together, these figures demonstrate the effectiveness of the PSM procedure.

### Association between DEX Use and Primary Outcome

As depicted in Table 3, the all-cause mortality rate over 7 days was 4.74% for those using DEX, a significant reduction compared to the 10.25% for non-user. According to univariable Cox regression, DEX use was significantly associated with mortality at 7 days (Table 3). The use of DEX was independently associated with a reduced risk of all-cause mortality, as confirmed by multivariable Cox regression that adjusted for relevant clinical factors. DEX administration was correlated with a significantly lower mortality rate at 7 days, both before (HR = 0.36, 95% CI: 0.19–0.68, *p* = 0.002) and after PSM (HR = 0.26, 95% CI: 0.12–0.54, *p* < 0.001; Model 3) (Table 4). Online supplementary Figure 3 illustrates that the Kaplan-Meier survival curves revealed a significantly higher 7-day survival rate for the DEX group in both unmatched and matched cohorts.

**Table 3.** Association between DEX administration and clinical outcomes among patients with IS-AF

Characteristics	Total	Non-Dex	Dex	HR (95% CI)	*p* value
*Before PSM*	*n = 1,355*	*n = 1,102*	*n = 253*	​	​
Primary outcome					
7-day mortality	125 (9.23%)	113 (10.25%)	12 (4.74%)	0.38 (0.21–0.68)	0.001
Secondary outcomes					
90-day mortality	483 (35.65%)	383 (34.75%)	100 (39.53%)	0.80 (0.64–0.99)	0.018
*After PSM*	*n = 416*	*n = 208*	*n = 208*	​	​
Primary outcome					
7-day mortality	40 (9.62%)	30 (14.42%)	10 (4.81%)	0.30 (0.15–0.61)	<0.001
Secondary outcomes					
90-day mortality	176 (42.31%)	96 (46.15%)	80 (38.46%)	0.71 (0.53–0.95)	0.023

PSM, propensity score matching; HR, hazard ratio; 95% CI, 95% confidence interval.

**Table 4.** Multivariate-adjusted HR (95% Cl) of DEX administration and clinical outcomes among patients with IS-AF

Categories	7-day mortality		90-day mortality	
	HR (95% CI)	*p* value	HR (95% CI)	*p* value
Before PSM				
Model 1	0.38 (0.21–0.68)	0.001	0.80 (0.64–0.99)	0.048
Model 2	0.30 (0.16–0.55)	<0.001	0.76 (0.59–0.96)	0.023
Model 3	0.36 (0.19–0.68)	0.002	0.84 (0.66–1.07)	0.162
After PSM				
Model 1	0.30 (0.15–0.61)	<0.001	0.71 (0.53–0.95)	0.023
Model 2	0.32 (0.15–0.65)	0.002	0.73 (0.54–0.98)	0.035
Model 3	0.26 (0.12–0.54)	<0.001	0.72 (0.53–0.97)	0.031

Model 1: crude.

Model 2: age, CCI, SAPSII, OASIS.

Model 3: age, CCI, SAPSII, OASIS, anti-platelet agents, anticoagulant drugs, thrombolysis.

SOFA, sequential organ failure assessment; SAPSII, simplified acute physiology score II; OASIS, oxford acute severity of illness score; PSM, propensity score matching; HR, hazard ratio; CI, confidence interval.

### Association between DEX Use and Secondary Outcomes

For those using DEX, the 90-day all-cause mortality rate was 39.53%, which is marginally above the 34.75% for non-users. Multivariable Cox models confirmed a significant association between DEX use and reduced 90-day mortality before (HR = 0.80, 95% CI: 0.64–0.99, *p* = 0.048) and after PSM (HR = 0.71, 95% CI: 0.53–0.95, *p* = 0.023)) (in Model 1). Model 3 showed that some associations were slightly reduced (before PSM, HR = 0.84, 95% CI: 0.66–1.07, *p* = 0.162; after PSM, HR = 0.72, 95% CI: 0.53–0.97, *p* = 0.031). Overall, the results imply that DEX could improve short-term survival in patients with IS-AF. This is corroborated by the Kaplan-Meier curves for 90-day survival (online suppl. Fig. 4).

### Subgroup Analysis

The protective effect of DEX was found to vary among different clinical and demographic groups, as indicated by subgroup analyses (online suppl. Table 4). DEX use was significantly associated with reduced mortality in patients aged 70 years or older (HR = 0.23, 95% CI: 0.09–0.58, *p* = 0.002), whereas this association was not significant in patients younger than 70 years (HR = 0.35, 95% CI: 0.08–1.04, *p* = 0.154). Both genders showed survival benefits, with females having HR = 0.18 (95% CI: 0.05–0.63, *p* = 0.008) and males HR = 0.33 (95% CI: 0.13–0.82, *p* = 0.017). DEX use was significantly associated with reduced mortality in patients without heart failure (HR = 0.13, 95% CI: 0.04–0.39, *p* < 0.001), without chronic kidney disease (HR = 0.28, 95% CI: 0.11–0.67, *p* = 0.004), and in patients with hypertension (HR = 0.11, 95% CI: 0.02–0.53, *p* = 0.006). Benefits were observed irrespective of diabetes or chronic pulmonary disease status. Regarding treatment-related factors, DEX demonstrated protective effects in patients not receiving CRRT (HR = 0.27, 95% CI: 0.13–0.60, *p* = 0.001) or vasopressors (HR = 0.17, 95% CI: 0.06–0.54, *p* = 0.002), as well as in patients receiving mechanical ventilation (HR = 0.25, 95% CI: 0.12–0.54, *p* < 0.001) or anticoagulation therapy (HR = 0.26, 95% CI: 0.12–0.56, *p* < 0.001). Survival benefits were observed with DEX use, regardless of whether antiplatelet therapy was administered. According to interaction analyses, individual factors did not significantly modify the DEX effect, implying consistent protective effects among multiple subgroups.

## Discussion

This study revealed that the administration of DEX was significantly associated with reduced short-term mortality in critically ill patients with ischemic stroke complicated by AF. After adjusting for confounding factors through PSM and both univariate and multivariable Cox regression analyses, DEX use was significantly associated with reduced all-cause mortality at both 7 days and 90 days. These observations suggest that DEX might contribute to improving short-term survival in this high-risk demographic. In specific subgroups, such as older patients, women, those with hypertension, and individuals without heart failure or chronic kidney disease, the survival benefit associated with DEX appeared to be more pronounced. While promising, our findings must be interpreted with caution due to the observational nature of the study. Our study demonstrates an association between DEX use and improved outcomes, but causality can only be established through randomized controlled trials.

To date, no studies have systematically evaluated the relationship between DEX use and mortality in IS-AF patients. Our study provides the first evidence supporting the potential survival benefit of DEX in this patient population. Previous ICU-based studies have indicated that DEX can reduce the incidence of new-onset AF and hospital mortality. For instance, a large propensity score-matched cohort study using the MIMIC-IV database reported that DEX significantly reduced the risk of new-onset AF (17.6% vs. 22.4%; HR = 0.80; 95% CI: 0.71–0.90) and in-hospital mortality (6.3% vs. 12.8%; HR = 0.43; 95% CI: 0.36–0.52) [21]. Perioperative studies, particularly in cardiac surgery, have consistently shown that DEX can effectively decrease postoperative AF incidence. In a meta-analysis of 18 randomized controlled trials comprising 2,933 patients, reported a significant reduction in postoperative AF (OR = 0.82; 95% CI: 0.69–0.98; *p* = 0.030) [22]. A prospective study also demonstrated that DEX is feasible and safe for acute ischemic stroke patients undergoing thrombectomy, providing effective sedation while maintaining stable respiratory function [23]. Furthermore, an ongoing multicenter RCT (PPDET, NCT04916197) is investigating the impact of 24-h post-recanalization DEX sedation on long-term outcomes [24]. Although prior studies mainly focus on surgical or ICU populations, our findings extend these observations to IS-AF patients.

Su et al. reported that low-dose DEX significantly reduced delirium and mortality in ICU patients, suggesting that therapeutic effects do not rely on high-dose administration [15]. Furthermore, in rhythm management, sympathetic inhibition by DEX at low doses may suffice to stabilize heart rhythm, reducing AF-related cardiac events. Jing et al. showed that non-loading DEX better maintained hemodynamic stability and anti-arrhythmic effects than loading doses in postoperative AF patient [22], which is particularly relevant for hemodynamically vulnerable IS-AF patients. Conversely, in certain inflammatory brain injury models, DEX dose-dependently reduced edema, neuronal apoptosis, and inflammatory mediator expression [25, 26], indicating that dose-response relationships may vary by pathological context. Our findings also suggest a potential early mortality signal for DEX administration of 24–72 h, although statistical significance was not reached due to wide confidence intervals. Overall, low-dose DEX may achieve maximal therapeutic benefit while maintaining safety in hemodynamically unstable populations. Future research should further elucidate dose-response relationships and optimize individualized regimens.

The underlying biological mechanisms remain incompletely elucidated but may involve several pathways. First, as a highly selective α2-adrenergic receptor agonist, DEX inhibits sympathetic activity and restores autonomic balance. AF patients often exhibit sympathetic overactivation; DEX may mitigate sympathovagal imbalance, reduce heart rate variability, and decrease arrhythmia occurrence, thereby stabilizing cardiac rhythm [27]. This is particularly relevant for ICU patients under stress. Second, DEX possesses anti-inflammatory and neuroprotective properties. Ischemic stroke triggers pro-inflammatory mediator release, neuronal apoptosis, axonal injury, and impaired regeneration. Controlling inflammation and apoptosis is critical for neuronal protection and recovery. Ischemic stroke is often accompanied by ischemia-reperfusion injury and inflammatory response of brain tissue. The release of inflammatory mediators such as TNF-α and IL-6 can aggravate cerebral edema and nerve damage [28–31]. DEX has been shown to downregulate p-JAK2 and p-STAT3, reducing neuroinflammation and apoptosis, which alleviates secondary brain injury and improves neurological outcomes [32–35]. Third, DEX helps maintain blood-brain barrier (BBB) integrity and improves cerebral microcirculation. Excessive inflammation after stroke can compromise the BBB and exacerbate neural injury [36–38]. DEX reduces inflammatory mediator expression and enhances tight junction protein expression, thus preserving BBB integrity [39, 40]. Finally, DEX sedation is superior to conventional sedatives in lowering ICU delirium incidence [41, 42], which is an independent risk factor for poor outcomes in stroke patients. Collectively, DEX may confer multi-mechanistic protection in IS-AF patients via autonomic modulation, anti-inflammation, neuroprotection, BBB stabilization, and cognitive function support.

Several limitations merit consideration. First, this study was retrospective and observational, based on the MIMIC-IV database; hence, the associations observed cannot establish a causal or definitive mortality benefit of DEX. Even with the use of PSM and multivariable Cox regression to balance measured baseline characteristics and adjust for potential confounders, residual confounding and selection bias may still be present. Specifically, the choice to give DEX was not random and could have been affected by the patientsʼ clinical state, comorbidities, hemodynamic condition, requirement for mechanical ventilation, and the sedation practices of the institution. Second, detailed information regarding DEX administration strategies, including cumulative exposure, infusion duration, and timing of initiation relative to stroke onset, was not available. Consequently, DEX exposure was considered a binary variable, which restricted the examination of potential dose-response relationships or effects over time. The lack of specific pharmacologic and temporal information hinders our exploration of whether the observed associations change with dosing intensity or timing of initiation. Third, despite the MIMIC-IV database reflecting real-world ICU practices and offering thorough mortality data, it fails to systematically capture standardized stroke-specific neurological severity measures before death. Significant variables like the National Institutes of Health Stroke Scale (NIHSS), the modified Rankin Scale, and structured evaluations of post-stroke functional status are not routinely recorded in a structured format. Consequently, these stroke-specific indicators could not be incorporated into baseline characterization or multivariable adjustment. Although multiple validated ICU severity scores, including SAPS II, SOFA, APS III, OASIS, GCS, and the Charlson Comorbidity Index, were included to account for overall illness burden and neurological status, these measures do not fully substitute for dedicated stroke severity scales. While mortality represents a clinically meaningful and objective endpoint, the absence of functional and neurological outcome measures limits our ability to evaluate the potential impact of DEX on post-stroke recovery trajectories. This distinction is particularly relevant in stroke populations, where survival and functional independence may diverge. Finally, the administration of DEX in ICU settings might suggest broader sedation and management strategies, rather than a direct pharmacological impact on mortality. In critically ill patients, treatment choices are affected by a range of complex and changing clinical factors, including the need for ventilation, hemodynamic status, and the sedation protocols of the institution. Even though we adjusted for several indicators of illness severity and organ support and used PSM to mitigate measured confounding, unmeasured aspects of ICU management could still influence the associations observed. Hence, the findings should be interpreted as associative, not causal, and the inherent residual confounding in real-world critical care practice cannot be entirely excluded. Further research that is prospective and multicenter in nature, with standardized treatment protocols, should include a detailed assessment of DEX dosage, timing, and duration, along with comprehensive adjustments for stroke severity and other relevant confounders. Ultimately, randomized controlled trials with adequate power are essential to verify the observed relationships and determine if DEX provides a real survival benefit for those with IS-AF.

## Conclusion

DEX administration was associated with reduced mortality in patients with ischemic stroke complicated by AF. These findings suggest that DEX may offer clinical benefits for this high-risk population. Considering the observational nature of this study, it is essential to perform large-scale, multicenter, and meticulously designed randomized controlled trials to confirm these findings and explore the underlying mechanisms.

## Statement of Ethics

In line with the Declaration of Helsinki, the MIMIC-IV database was developed and approved by the Institutional Review Board of Beth Israel Deaconess Medical Center (Approval No. 2001P-001699/14). The present study used publicly available, de-identified MIMIC-IV data for secondary analysis. This study did not require additional ethical approval because it involved only secondary analysis of anonymized data, with no direct participant interaction or access to identifiable information.

## Conflict of Interest Statement

No conflicts of interest were reported by the authors.

## Funding Sources

The authors declare no funding was obtained or used for this study.

## Author Contributions

Data curation, data analysis, and manuscript preparation were performed by S.K., L.H.W., Y.Z.N., and X.S.Q. B.Z.Y. made substantial contributions to the additional analyses and manuscript revision during the revision process. L.H.W. and Y.Z.N. were responsible for language and grammar checking. The study design and manuscript revision were contributed to by B.Z.Y., S.K., and X.S.Q. B.Z.Y. provided methodological guidance, supervised the revision process, contributed to the interpretation of the additional analyses, and critically revised the manuscript. B.Z.Y. coordinated the preparation and revision of the manuscript and the responses to the reviewers. All authors reviewed and approved the final manuscript and agree to be accountable for the content of the work.

## Data Availability

This studyʼs analyzed data are obtainable from the MIMIC-IV database through PhysioNet (https://mimic.mit.edu/docs/iv/), with access contingent upon credentialed approval. Further inquiries regarding the data and related materials can be directed to the corresponding author.
